# Editorial: Advances in crop resistance breeding using modern genomic tools

**DOI:** 10.3389/fpls.2023.1143689

**Published:** 2023-02-16

**Authors:** Lin Huang, Yinghui Li, Shisheng Chen, Sambasivam Periyannan, Tzion Fahima

**Affiliations:** ^1^ State Key Laboratory of Crop Gene Exploration and Utilization in Southwest China, Sichuan Agricultural University, Chengdu, China; ^2^ Triticeae Research Institute, Sichuan Agricultural University, Chengdu, China; ^3^ Institute of Evolution, University of Haifa, Haifa, Israel; ^4^ Peking University Institute of Advanced Agricultural Sciences, Weifang, Shandong, China; ^5^ The University of Southern Queensland, School of Agriculture and Environmental Science, Centre for Crop Health, Toowoomba, QLD, Australia

**Keywords:** crop species, plant pathogens, disease resistance, genomic tools, resistance breeding

Plant diseases constitute a major threat to global crop production and food security. Plants respond to pathogens using a two-tier innate immune system triggered by both cell-surface-localized pattern-recognition receptors (PRRs) and intracellular nucleotide-binding leucine-rich repeat receptors (NLRs) (reviewed by Zhou and Zhang, 2020; Ngou et al., 2022). The deployment of immune receptors to breed disease-resistant cultivars is an effective and sustainable approach to controlling crop diseases. However, it largely relies on the ability to identify and transfer novel and useful resistance (*R*) genes rapidly from the source to commercial crop varieties. Over the last two decades, with advances in DNA sequencing, molecular marker, and genotyping techniques, remarkable progress has been made in the identification of *R*-genes both from crop species and their wild relatives. Subsequently, novel strategies have been implemented through the in-depth understanding of the *R*-gene-mediated resistance mechanisms and the ability to transfer R-genes rapidly into commercial cultivars.

This Research Topic on “Advances in Crop Resistance Breeding using Modern Genomic Tools” aims to explore the application of modern genomic tools to characterize genetic loci associated with disease resistance and to accelerate the generation of disease resistant cultivars. The collection consists of five articles covering techniques such as RNAi technology, next-generation sequencing, and comparative genomics.

The review article by Halder et al. describes application of RNA interference (RNAi) and clustered regularly interspaced short palindromic repeats (CRISPR/Cas) based genome-editing strategy to induce disease resistance. Both technologies are powerful tools to regulate gene expression and to introduce pest and disease resistance in crops. In addition to their broad applications enabling creation of resistant crops against bacteria, fungal, viral pathogens and insect pest, the authors also summarized the commercial products released to date and discussed public awareness as well as the necessity to incorporate these innovative strategies in the integrated pest/disease management.

Advances in next-generation sequencing (NGS) platforms coupled with improved genome assembly algorithms have caused a surge in whole-genome reference sequences of many crops during the past decade. The genomic information of crops has enabled the comprehensive study of disease-resistance related genes evolution, diversity and their functions. For instance, Zhu et al. identified 102 *WRKY* genes, which play a lead role in biotic and abiotic stress tolerance, from the cassava (*Manihot esculenta* Crantz) genome by performing a whole-genome scan using conserved domains of the WRKY gene family. Six *MeWRKY IIas* transcripts were found to be significantly up-regulated by SA (salicylic acid), MeJA (methyl jasmonate) and *Xam* (*Xanthomonas axonopodis* pv. *Manihotis*) treatment. *MeWRKY27* and *MeWRKY3*3 were confirmed as essential regulators of cassava against *Xam* infection and considered as the target genes for resistance to CBB (*Cassava* bacterial blight). Zuo et al. predicted 130 legume lectin (*LegLu*) genes in *Brassica napus* using Darmor-bzh v4.1 genome sequence information. Among them, 40 *BnLegLu* genes showed strong response to *Sclerotinia sclerotiorum* (SD) infection and four were from the SD resistance locus as predicted through a genome-wide association analysis (GWAS). Further, *BnLegLu*-16 gene’s role in SD resistance was confirmed through transient expression in tobacco.

*NLRs* are among the major source of *R*-genes for improvement of crop resistance against diseases. With the availability of pan-genome references, haplotypic analysis of *NLRs* across multiple accessions provides valuable insights into the evolutionary aspects of *NLRs* and also enables fast detection of novel *R*-genes. Si et al. characterized and compared *NLR* type *R*-genes across the whole genomes of four *Ipomoea* species (*I. batatas*, *I. trifida, I. triloba*, and *I. nil*) and described collinearity, cluster, and duplication events at the *NLR* locus. Subsequently, based on sequence comparisons of transcriptomic data of resistant and susceptible cultivars of sweet potato, 11 and 19 *NLR*-encoding genes were identified as candidates for resistance to stem nematodes and *Ceratocystis fimbriata*, respectively. Hence increased sequencing of diverse accessions of crop species has accelerated *R*-gene identification for breeding nematode-resistant sweet potatoes.

With the reduction in sequencing costs, NGS-based trait mapping approaches become more common in crop improvement. NGS technologies combined with robust phenotyping accelerate marker-trait associations studies and candidate genes identification for resistance. In this Research Topic, Feng et al. detected quantitative trait loci (QTLs) for *Fusarium verticillioides* resistance on the basis of 10-fold coverage genome resequencing data and resistance phenotype of three maize populations which consisted of 450 progenies with teosinte gene introgression. Interestingly, two of the QTLs overlap with yield related traits; thus, they might ensure yield stability while exerting *Fusarium* ear-rot resistance in maize.

Overall, this collection highlights the impact of latest genomic tools and technologies on the identification, characterization, and utilization of genetic resistance components to accelerate crop breeding for disease resistance. Tools such as next generation sequencing, multi-omics, high-density genotyping, and genome editing will be used more frequently in coming years; thereby, cultivars with inbuilt genetic resistance will come to play significant roles in mitigating food security threats posed by rapidly evolving crop pathogens.

## Author contributions

All authors listed have made a substantial, direct, and intellectual contribution to the work and approved it for publication.
